# Aggregation kinetic dataset to determine the stability of the purified and refolded recombinant ppTvCP4 protein of *Trichomonas vaginalis*

**DOI:** 10.1016/j.dib.2016.05.066

**Published:** 2016-06-02

**Authors:** Rosa E. Cárdenas-Guerra, Jaime Ortega-López, Rossana Arroyo

**Affiliations:** a Departamento de Infectómica y Patogénesis Molecular, Centro de Investigación y de Estudios Avanzados del Instituto Politécnico Nacional (CINVESTAV-IPN), Av. IPN # 2508, Col. San Pedro Zacatenco, Delg. Gustavo A. Madero, CP 07360 México, D.F., Mexico; b Departamento de Biotecnología y Bioingeniería, Centro de Investigación y de Estudios Avanzados del Instituto Politécnico Nacional (CINVESTAV-IPN), Av. IPN # 2508, Col. San Pedro Zacatenco, Delg. Gustavo A. Madero, CP 07360 México, D.F., Mexico

**Keywords:** PpTvCP4r, Buffer exchange, Protein aggregation, *Trichomonas vaginalis*

## Abstract

The recombinant ppTvCP4 (ppTvCP4r) protein, a specific inhibitor of the proteolytic activity and virulence properties of *Trichomonas vaginalis,* depending on cathepsin L-like cysteine proteinases (CPs) (http:dx.doi.org/ 10.1016/j.biocel.2014.12.001[1], http:dx.doi.org/ 10.1016/j.micinf.2013.09.002[2], http:dx.doi.org/ 10.1155/2015/946787[3]) was stable in the elution buffer up to two months at 4 °C. However, it was prone to aggregate in PBS (functional assay buffer) [1]. Therefore, before functional assays, the aggregation kinetic of refolded ppTvCP4r was determined after the exchange to PBS. Samples of purified and refolded ppTvCP4r (0.15 mg/ml) in PBS were incubated for 0–24 h at 4 and 25 °C, spun down, measured the protein concentration in the supernatant and checked for the presence of aggregated protein in the pellet. The concentration of protein progressively decreased in the supernatant through time at both temperatures as the protein aggregated. Data in this article are related to the research paper [1].

## Specifications Table

**Table:** 

Subject area	Biochemistry
More specific subject area	Protein aggregation kinetic
Type of data	Figure
How data was acquired	A NanoDrop 2000 was used to determine protein concentration of samples at 280 nm
Data format	Analyzed data
Experimental factors	The purified and refolded recombinant ppTvCP4 protein buffer was exchanged to PBS by using a PD10 desalting column
Experimental features	To determine the protein aggregation of ppTvCP4r in PBS, the recombinant protein was incubated at 4 and 25 °C, centrifuged, and protein concentration at 280 nm in the supernatant was determined
Data source location	Mexico City, Mexico
Data accessibility	The data are with this article

## Value of the Data

• A simple experiment helps to indicate whether a protein of interest in a particular buffer, temperature, and protein concentration could be aggregated.
• The ppTvCP4r protein in the first four hours did not show aggregation. However, after 5 h there was a slow aggregation process of ppTvCP4r in PBS at both temperatures tested.
• Although the conditions shown in here applied to ppTvCP4r, a similar procedure could be applied and adapted to any protein by modifying buffer, protein concentration, temperature, and time, according to the particular conditions of the protein and assay of interest.

## 1 Data

An aggregation kinetic of ppTvCP4r in PBS was performed at different times and temperatures, centrifugated and protein concentration in the supernatant was measured. The presence of a pellet indicated protein aggregation.

## 2 Experimental design, materials and methods

### 2.1 Buffer exchange of the ppTvCP4r protein

Fractions containing the purified and refolded ppTvCP4r protein in the elution and refolded buffer (20 mM Tris–HCl [pH 8.0], 0.5 M NaCl, 0.5 M imidazole, and 1 M urea) were pooled and stored at 4 °C. Previous to any functional assays [1], [2], the buffer of the purified ppTvCP4r protein was exchanged to PBS by using a PD10 desalting column (GE Healthcare, CA, USA), following a procedure recommended by the provider as previously described [1]. Briefly, a 2.5 ml of the purified and refolded ppTvCP4r protein (0.1–0.2 mg/ml) was applied to a 10 ml PD10 column pre-equilibrated with PBS (pH 7.4). The protein was eluted with 3.5 ml of PBS.

### 2.2 Aggregation kinetic

**Fig. 1:**
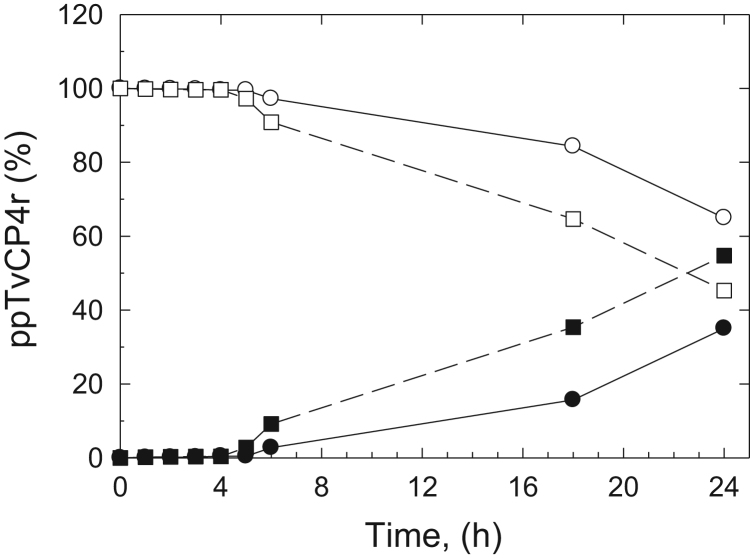
Data of the ppTvCP4r aggregation in PBS. The aggregation kinetic of ppTvCP4r (0.15 mg/ml) in PBS incubated at 4 °C (circles) and 25 °C (squares) for 24 h. Triplicated samples were taken at different time-points, centrifuged and protein concentration in the supernatant was determined. The % of the soluble ppTvCP4r (empty symbols) was determined by taking the initial absorbance at 280 nm as 100% (time zero). The % of aggregation (filled symbols) was estimated from the difference (100% soluble ppTvCP4r). A visible protein pellet was observed only after 18 h incubation. The standard deviation for each triplicate sample time-point was less than 0.1%.

The aggregation kinetic of refolded ppTvCP4r in PBS was determined. Samples of ppTvCP4r (0.15 mg/ml) in PBS were incubated for 0–24 h at 4 and 25 °C in a 1.5 ml microfuge tubes. Triplicated samples were taken at different time-points (0, 4, 6, 18, 24 h) and were spun down at 13 000*g* for 5 min. The protein concentration in the supernatants was measured at 280 nm in the NanoDrop 2000 (Thermo Scientific, USA) and the pellet was checked for the presence of aggregated protein. The % of the soluble ppTvCP4r protein was determined by taking the initial absorbance at 280 nm as 100% (time zero). The % of protein aggregation was estimated from the difference (100% soluble ppTvCP4r). The standard deviation for each triplicate sample time-point was less than 0.1% [1]. The concentration of protein progressively decreased in the supernatant through time at both temperatures as the protein aggregated (Fig. 1).
